# Rare squamous cell carcinoma arising from a presacral epidermoid cyst: A case report

**DOI:** 10.1016/j.ijscr.2019.12.022

**Published:** 2019-12-19

**Authors:** Manato Ohsawa, Tetsuya Kagawa, Ryoji Ochiai, Naruyuki Kobayashi, Shinji Hato, Isao Nozaki, Hiroyuki Takahata, Norihiro Teramoto, Takaya Kobatake

**Affiliations:** a Departments of Surgery, National Hospital Organization Shikoku Cancer Center, 160 Minamiumemotomachikou, Matsuyama-shi, Ehime, Japan; b Departments of Pathology, National Hospital Organization Shikoku Cancer Center, 160 Minamiumemotomachikou, Matsuyama-shi, Ehime, Japan

**Keywords:** Presacral epidermoid cysts, Benign cysts, Fetal period, Squamous cell carcinoma

## Abstract

**Introduction:**

Presacral epidermoid cysts are uncommon, usually benign cysts caused by developmental abnormalities in the fetal period. We present a rare case of squamous cell carcinoma arising from a presacral epidermoid cyst.

**Presentation of case:**

A 59-year-old woman complained of tenesmus and discomfort in the buttocks. Computed tomography revealed a 50-mm well-defined cystic mass in the presacrum and a 70-mm solid mass extending from the cyst into the rectum, vagina, and left sciatic spine. On T1-weighted magnetic resonance images, the cyst was unilocular and the mass was marginated with low intensity. On T2-weighted images, the mass had high intensity. A malignant presacral developmental cyst was diagnosed, without obvious metastasis. Using abdominal and parasacral approaches, Hartmann’s operation was performed with multiorgan resection, including the sacrum, coccyx, left sciatic spine, internal obturator muscle, rectum, and uterine appendage. Histopathology of the excised specimen revealed a squamous cell carcinoma originating from the presacral epidermoid cyst.

**Discussion:**

Reports of malignant transformation of epidermoid cysts in the presacral space, as in the present case, are extremely rare. Because of their unusual location and slow growth, epidermoid cysts tend to remain asymptomatic. Because the patient had a malignant tumor with suspected invasion of adjacent organs, combination surgery was selected.

**Conclusion:**

Although further research is required, presacral epidermoid cysts are extremely rare and may be malignant. Thorough preoperative image evaluation is crucial for complete resection.

## Highlights

• Presacral epidermoid cysts are extremely rare and require further study.
• Thorough preoperative imaging evaluation is important for complete resection.
• Multidisciplinary treatments may be effective.
• Presacral epidermoid cysts may be malignant.

## 1 Introduction

The present work has been reported in line with the SCAREcriteria [1]. Presacral epidermoid cysts are caused by developmental abnormalities in the fetal period, as are dermoid cysts and tailgut cysts [2]. Reports of malignant presacral epidermoid cysts are extremely rare despite their malignant potential [[3], [4], [5]]. Herein, we present a rare case of a squamous cell carcinoma originating from a presacral epidermoid cyst.

## 2 Presentation of case

A 59-year-old woman consulted a primary care doctor with the chief complaints of tenesmus and discomfort in the buttocks. A rectal submucosal tumor was suspected after colonoscopy, and she was referred to our hospital. She had no past medical or surgical history; however, her brother had rectal cancer. On physical examination, her abdomen was soft and flat, with no tenderness.

**Fig. 1:**
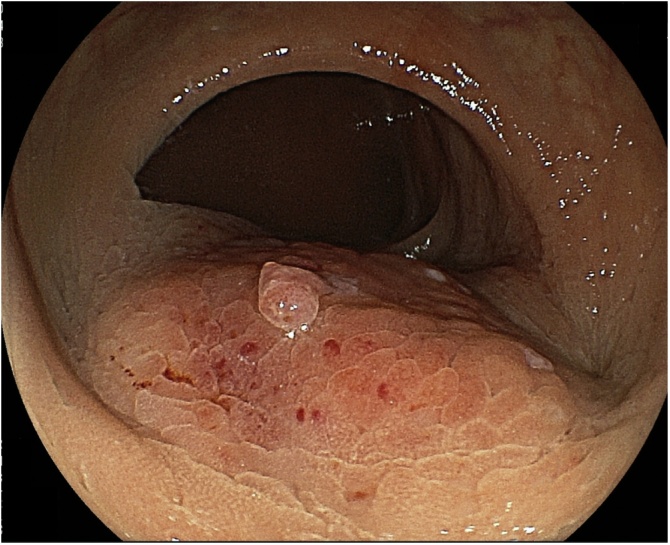
Colorectal examination shows exudation from the outside rectal wall, and a rectal submucosal tumor was suspected. No erosion or ulceration is seen on the mucosal surface.

Biochemistry tests revealed elevated levels of squamous cell carcinoma-related antigen (26.4 ng/mL) and carbohydrate antigen 125 (40.5 U/mL). Levels of carcinoembryonic antigen, carbohydrate antigen 19-9, carbohydrate antigen 72-4, and alpha-fetoprotein were within normal limits. Colorectal examination showed exudation from outside the rectal wall, consistent with a rectal submucosal tumor (Fig. 1). There was no erosion or ulceration on the mucosal surface.

**Fig. 2:**
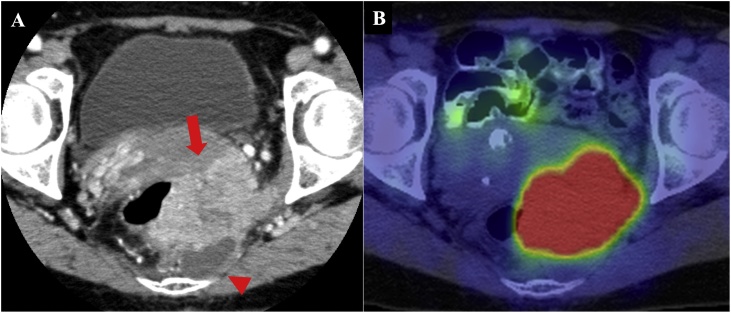
Contrast computed tomography reveals a 50-mm well-defined cystic mass in the presacrum (arrowhead) and a 70-mm solid mass extending from the cyst into the rectum, vagina, and left sciatic spine (arrow) (A). The solid mass demonstrates high fluorodeoxyglucose uptake on positron emission tomography (B).

**Fig. 3:**
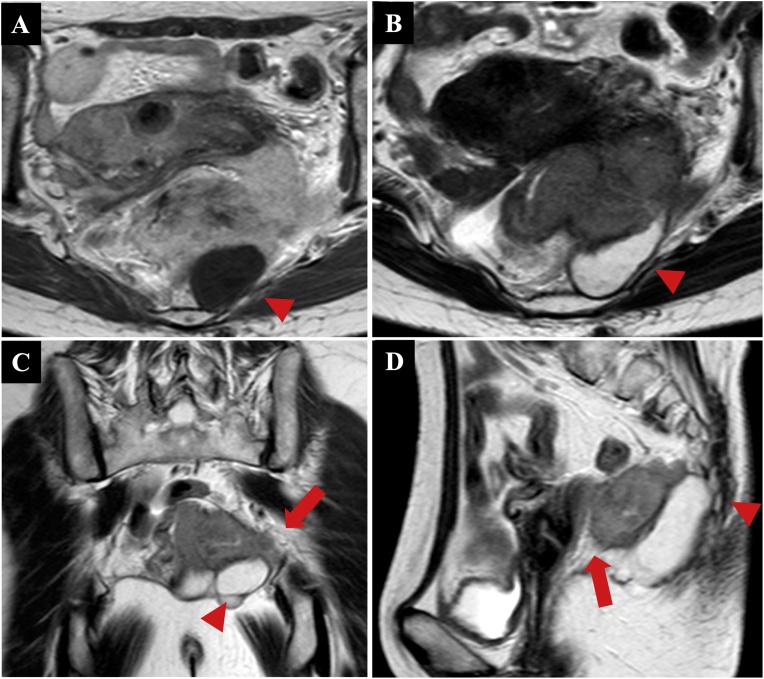
T1-weighted magnetic resonance image shows a unilocular cyst and marginated low-intensity mass (arrowhead) (A). T2-weighted magnetic resonance image shows a high-intensity mass (arrowhead) (B). A solid mass with a contrast effect is found on the left side and head side of the cyst in the T2-weighted magnetic resonance image. The solid part touches the rectum, vagina, and left sciatic spine, with possible invasion (arrow). The cyst does not involve the levator ani muscle in the coccyx and sacrum (arrowhead) (C: Coronal section, D: Sagittal section).

Contrast computed tomography revealed a 50-mm well-defined cyst in the presacrum and a 70-mm solid mass extending from the cyst into the rectum, vagina, and left sciatic spine. There was no metastasis of the lymph nodes (Fig. 2A). The solid mass demonstrated high fluorodeoxyglucose uptake on positron emission tomography (Fig. 2B). On T1-weighted magnetic resonance imaging, the cyst was unilocular and the mass was marginated with low intensity (Fig. 3A); on T2-weighted imaging, the mass had high intensity (Fig. 3B). A solid mass with a contrast effect was found on the left and head sides of the cyst. The mass touched the rectum, vagina, and left sciatic spine, with possible invasion. The cyst did not involve the levator ani muscle, which extends from the coccyx to the sacrum (Fig. 3C, D).

Based on the above findings, the patient was diagnosed with a malignant presacral developmental cyst. There was no obvious metastasis, and surgical management was adopted. Using the abdominal and parasacral approaches, Hartmann’s operation (rectal excision without anastomosis) was performed with multiple organ resection including the sacrum, coccyx, left sciatic spine, internal obturator muscle, rectum, and uterine appendage. The sacrum was dissected at the S4 level. A gynecologist and an orthopedic surgeon performed the complete resection. The operative time was approximately 842 min, and intraoperative blood loss was approximately 4,210 mL.

**Fig. 4:**
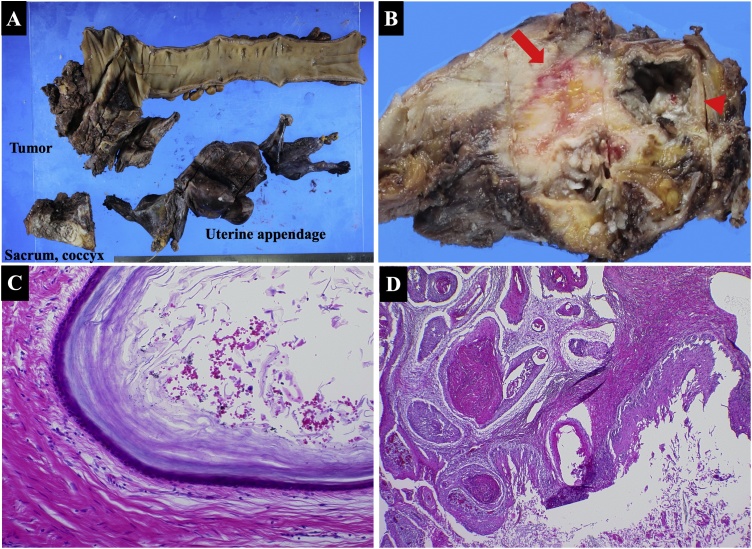
Macroscopic examination. Resected rectum including the tumor, sacrum, coccyx, and uterine appendage (A). Macroscopic examination of the resected specimen reveals a tumor composed of a 50-mm cyst (arrowhead) and an 80-mm solid lesion (arrow) in the front of the sacrum and coccyx. No skin appendages are seen in the cyst. The cut surface of the solid lesion reveals grayish white tissue (B). Microscopic examination. The luminal surface of the cyst is covered with squamous epithelium (hematoxylin-eosin stain, ×200 magnification) (C). Continuous invasion and growth of the squamous cell carcinoma seen from the strong atypical cells of cysts to the solid part (hematoxylin-eosin staining, ×100) (D).

We removed the rectum including the tumor, sacrum, coccyx, and uterine appendage (Fig. 4A). Macroscopic examination of the resected specimen revealed a tumor composed of a 50-mm cyst and an 80-mm solid lesion in front of the sacrum and coccyx (Fig. 4B). There were no skin appendages in the cyst. The cut surface of the solid lesion revealed grayish white tissue.

Microscopic examination demonstrated that the luminal surface of the cyst was covered with squamous epithelium (Fig. 4C), with continuous invasion and growth of the squamous cell carcinoma from the strongly atypical cells of cysts to the solid part (Fig. 4D). Consequently, the patient was diagnosed with squamous cell carcinoma arising from a presacral epidermoid cyst. No malignant cells were detected in the resection stump.

The patient recovered uneventfully and was discharged 39 days after surgery. The preoperative symptoms of tenesmus and discomfort in the buttocks disappeared. No recurrence was found in the first follow-up computed tomography 3 months after surgery. However, 7 months after surgery, computed tomography showed recurrence of the metastasis in the pelvic lymph nodes. Because the patient refused therapeutic intervention, best supportive care was adopted. She died 10 months after the surgery. No autopsy was performed.

## 3 Discussion

Presacral tumors are uncommon, which occurs in 1 of 40,000–63,000 patients, and 60 % of presacral tumors are congenital [6]. “Developmental cysts” are defined as presacral congenital cystic tumors caused by a developmental abnormality in the embryonal phase and are considered to originate from caudal embryonic vestiges [2]. Developmental cysts occur mostly in middle-aged women [7] and are pathologically classified as epidermoid, dermoid, or tailgut cysts. Both epidermoid and dermoid cysts are lined with stratified squamous epithelia; however, dermoid cysts also contain skin appendages. Tailgut cysts are lined with various types of epithelial cells, such as columnar, squamous, and transitional cells [2].

The reported incidence rate of malignant tumors arising from epidermoid cysts is 0.011 %–2.2 % [8,9]. The exact mechanisms whereby the epidermoid cysts become malignant remains unclear; however, it may be attributed to the chronic inflammatory responses to repeated cyst ruptures and subtotal resection of the cyst wall [10]. Most of the reported squamous cell carcinomas originated from epidermoid cysts in the brain, liver, or skin [[11], [12], [13]]. Reports of malignant transformation of epidermoid cysts in the presacral space, as in the present case, are extremely rare; in fact, a PubMed search of English articles yielded only three results [[3], [4], [5]]. Because of their unusual location and slow growth, epidermoid cysts tend to remain asymptomatic. Pain accompanying presacral lesions has been associated with secondary infection and malignant degeneration [4].

Computed tomography and magnetic resonance imaging are useful for diagnosing presacral tumors. They have low-signal intensity on T1-weighted magnetic resonance images and high-signal intensity on T2-weighted images. Epidermoid cysts contain fatty elements, such as desquamated debris, cholesterol, keratin, and water [14].

Preoperative biopsy should not be performed in patients with epidermoid cysts because it can lead to tumor dissemination, abscess, fecal fistula, or meningitis [6]. Malignant transformation must be considered in the differential diagnosis when there is focal wall thickening of the cyst on contrast-enhanced imaging [4]. Complete surgical excision is recommended as a diagnostic treatment. Epidermoid tumors are located deep within the pelvis and hence are difficult to reach; therefore, they are traditionally accessed through a sacral, abdominal, or combined abdomino-sacral approach [15]. The best approach is still controversial. Recently, a successful laparoscopic approach has been reported [16]. Selection of the surgical approach should consider tumor size, tumor invasion of other organs, and whether the tumor is malignant or benign [17]. In the present case, because the patient had a malignant tumor with suspected invasion of adjacent organs, combination surgery was selected.

There is still no definitive policy on adjuvant or recurrent treatment for epidermoid tumors, and further research is needed. Radiation therapy and chemotherapy may be useful, as they have been shown to prolong survival [18,19]. In our case, pelvic lymph node metastasis recurred 7 months after surgery. Additional therapeutic intervention was not desired by the patient; hence, it was not provided. However, it may be necessary to consider multidisciplinary treatments for malignant transformation in cases such as the one presented here.

## 4 Conclusion

Presacral epidermoid cysts are rare and require further study. Owing to their malignant potential, thorough preoperative imaging evaluation is important for complete resection. Multidisciplinary treatments may be effective.

## Sources of funding

The authors declare that this study was not funded externally.

## Ethical approval

As a case report without Protected Health Information, no ethics approval was required for this project.

## Consent

Written informed consent was obtained from the patient for the publication of this case report and any accompanying images. A copy of the written consent is available for review by the Editor-in-Chief of this journal.

## Author contribution

MO and TK drafted the article and performed the literature search. MO, TK, RO, NK, SH, IN, HT, NT, and TK contributed to patient care and participated in the critical revision of the article. All authors have read and approved the final article.

## Registration of research studies

This is a case report.

## Guarantor

Manato Ohsawa.

## Provenance and peer review

Not commissioned, externally peer-reviewed.

## Declaration of Competing Interest

The authors declare no conflicts of interest.
